# Knowing the gap: medication use, adherence and blood pressure control among patients with hypertension in Indonesian primary care settings

**DOI:** 10.7717/peerj.13171

**Published:** 2022-03-25

**Authors:** Adji Prayitno Setiadi, Anita Febriandini, Eltia Trinanda, Wiweka Aryaguna, Irene Mutho’atin Chusna, Yulia Nurlaili, Bruce Sunderland, Yosi Irawati Wibowo

**Affiliations:** 1 Centre for Medicines Information and Pharmaceutical Care (CMIPC), Faculty of Pharmacy, Universitas Surabaya, Surabaya, East Java, Indonesia; 2 Faculty of Pharmacy, Universitas Surabaya, Surabaya, East Java, Indonesia; 3 Medical School, Faculty of Health Sciences, Curtin University, Perth, Western Australia, Australia

**Keywords:** Hypertension, Drug therapy, Adherence, Lifestyle behaviour, Developing country, Primary care, Blood pressure control

## Abstract

**Background:**

Hypertension is a major risk factor for global disease burden, however, little is known regarding the profiles of patients with hypertension in Indonesian primary care settings.

**Objective:**

This study aimed to profile medication use, adherence to medications and lifestyle modifications as well as blood pressure control among patients with hypertension in Indonesian primary health centres (PHCs).

**Methods:**

A cross-sectional study design used a structured data collection tool (questionnaire and checklist). Patients aged ≥18 years with a diagnosis of hypertension, and prescribed an antihypertensive medication, and attending follow-up visits in the five PHCs in Surabaya, Indonesia, during a two-week study period (May–October 2019) were included. Descriptive analyses summarised the data, while binary logistic regression provided any independent associations between adherence profiles and blood pressure control.

**Results:**

Of 457 eligible patients, 276 patients consented: PHC A (*n* = 50/91), PHC B (*n* = 65/116), PHC C (*n* = 47/61), PHC D (*n* = 60/88), PHC E (*n* = 54/101), giving an overall response rate of 60.4%. Patients were mainly treated with a single antihypertensive medication, *i.e.*, amlodipine (89.1%), and many had not achieved blood pressure targets (68.1%). A majority reported notable levels of non-adherence to medication (low/intermediate, 65.2%) and poor healthy lifestyle behaviours, particularly physical activity (inadequate, 87.7%) and discretionary salt use (regularly, 50.4%). Significant associations were found between low medication adherence, discretionary salt use and smoking, with blood pressure control.

**Conclusions:**

The study findings provide the evidence needed to improve the current level of sub-optimal blood pressure management among patients with hypertension in these Indonesian primary care settings. Particular emphasis should be placed on antihypertensive medication adherence and healthy lifestyle behaviours through locally tailored hypertension-related interventions.

## Introduction

Hypertension is a major risk factor contributing to disease and economic burdens in South-east Asia, including Indonesia (Ng et al., 2014; Endarti et al., 2021). The prevalence of hypertension among Indonesians has increased from 26.5% in 2013 to 34.1% in 2018 (National Institute of Health Research and Development, Ministry of Health Republic of Indonesia, 2019; National Institute of Health Research and Development, Ministry of Health Republic of Indonesia, 2013). In addition to this increment, other hypertension-linked risk factors, such as unhealthy eating, physical inactivity and obesity have been reported as rising among Indonesians (National Institute of Health Research and Development, Ministry of Health Republic of Indonesia, 2019). These factors contribute to the notable increase of cardiovascular disease. Based on Baseline Health Research 2014, stroke and ischaemic heart disease ranked respectively as the first and second leading causes of death in the adult population (21.1% and 12.9%, respectively) (Ministry of Health Republic of Indonesia, 2014). Hence, the Indonesian Government has included hypertension control as a national health indicator (The Government of Republic of Indonesia Government, 2015).

A systematic review which included publications from a range of developed and developing countries reported that overall 45.2% of hypertensive patients were non-adherent to their medications. A very high proportion (83.7%) of patients with uncontrolled hypertension were also non-adherent. Notably, 62.5% of African and Asians were medication non-adherent (Abegaz et al., 2017). A recent review has noted the downward trend in hypertension prevalence in Western industrial countries but an increasing trend in developing countries. Increasing urbanization and a shift towards Western diet are acknowledged for this trend (Ibrahim, 2018). Contributing factors included excessive salt and alcohol consumption, lower fruit and vegetable intake, physical inactivity and obesity. In addition, a reported tendency existed between low birth weight and adult hypertension (Ibrahim, 2018). Low adherence to treatment raises the issues of treatment cost effectiveness. A recent review has reported wide variation in the cost per mm Hg reduction from a range of interventions, both within and across countries. Individual studies indicated that treatment was more cost-effective when applied to populations at higher CVD risk (Kostova et al., 2020).

The management of hypertension and related risk factors are addressed through adherence to lifestyle modifications and drug therapy when indicated (Williams et al., 2018; Indonesian Society of Hypertension, 2019). Lifestyle requirements include adequate physical activity, a healthy diet, reducing alcohol consumption, not smoking and keeping a normal body weight; all have been associated with controlled blood pressure (Williams et al., 2018; Dickinson et al., 2006). Five major classes of antihypertensive medications are recommended for managing patients: ACE inhibitors (ACEI), angiotensin receptor blockers (ARB), calcium channel blockers (CCB), beta-receptor blockers (BB) and thiazides (Williams et al., 2018). The 2018 European Society of Cardiology/ European Society of Hypertension (ESC/ESH) guidelines for the management of hypertension, which were adopted by the Indonesian Society of Hypertension (InaSH)’ consensus in 2019, preferred a two-drug combination; however, monotherapy has been recommended for low risk patients with stage-1 hypertension, or very high-risk patients with high-normal BP, or frail elderly (Williams et al., 2018; Indonesian Society of Hypertension, 2019).

The Indonesian healthcare system has seen the introduction of a national health insurance scheme, the *Jaminan Kesehatan Nasional* (JKN), in 2014. It aimed to provide access to basic health care for the entire population. This scheme implemented a referral system in which primary care facilities function as a gatekeeper to secondary/tertiary care (Health and Social Security Agency, 2014). *Puskesmas* (Primary Health Centres, PHCs) are functional units of a Regional Health Office to provide primary care at district level (*kecamatan*). The Indonesian Government has increased the number of PHCs to 10,134 (with a ratio of 1.4 per district) in 2019 (Ministry of Health Republic of Indonesia, 2020). Thus, PHCs could be seen as one of the largest public sector initiatives resulting in high numbers of patients, providing adequate facilities, manpower and significant financing.

PHCs play a key role in the management of patients with chronic hypertension. Drugs used in PHCs should be based on the National Formulary which in 2017 includes antihypertensive agents of classes CCB (*i.e.*, amlodipine, nifedipine, diltiazem), BB (*i.e.*, atenolol), ACEI (*i.e.*, captopril), and thiazide (*i.e.*, hydrochlorothiazide) (Ministry of Health Republic of Indonesia, 2013). Locally based small studies in Indonesian primary care settings have explored drug therapy and adherence among patients with hypertension reporting low levels of medication adherence, ranging from 20–59%  (Liberty, Pariyana & Roflin, 2017; Sinuraya et al., 2018; Setiadi et al., 2021; Rahmadani & Sari, 2018). However, in addition to blood pressure control with medications, its management should consider related risk factors and adherence to lifestyle modifications. In particular, the new 2019 InaSH consensus has recommended a lower target for optimum blood pressure, *i.e.*, a systolic pressure of ≤130 mmHg and a diastolic pressure of 70–79 mmHg (Indonesian Society of Hypertension, 2019). Therefore, this study aimed to profile medication use, adherence to medications and lifestyle modifications as well as blood pressure control among patients with hypertension in Indonesian PHCs.

## Materials & Methods

The study was approved by the Ethics Committee of the University of Surabaya (No: 082/KE/VII/2019).

### Study setting

This study was conducted at five PHCs in Surabaya, Indonesia, as follows: PHC (A), PHC (B), PHC (C), PHC (D) and PHC (E). Surabaya is the capital city of East Java Province and the second largest city in Indonesia. Surabaya covers 31 districts, and has 63 PHCs with a ratio of 0.22 per 1,000 population (Surabaya City Health Office, 2019). The five PHCs used as study settings had an approximately 500 to 1,200 outpatients per fortnight, with approximately 100 patients with hypertension (based on information from Surabaya Health Office); it should be noted that PHC patients were generally provided with a two-week to one-month’s medication supply.

### Study design and sampling

This was a descriptive cross-sectional study which included a census sample of patients with hypertension from each PHC during a two-week period. Patients aged ≥18 years with a diagnosis of hypertension (*i.e.*, physician diagnosed as recorded in the patient records), prescribed an antihypertensive medication, and attended for follow-up visits were included. Pregnant women and patients with mental illnesses were excluded from this study. During the study period, the PHCs provided services to 457 eligible patients, using 95% confidence interval and β = 0.8 and 5% margin of error, a minimum sample size of 209 was considered adequate (Lwanga & Lemeshow, 1991).

### Data collection

A structured data collection tool that included a questionnaire (to interview a patient) and a checklist (to extract necessary information from patient records or prescriptions) was used. The following data were collected:

*Patient characteristics.* Sociodemographic data (specifically age, gender, education attainment, occupation and income) were obtained using the questionnaire (Section A). While data on the clinical factors (*i.e.*, duration of hypertension, family history of hypertension, and the related vascular diseases) were derived from patient records.

*Antihypertensive drug use*. Information on the patient’s prescribed antihypertensive drugs was derived from the patient prescription.

*Adherence to antihypertensive medications*. Medication adherence over the previous month was determined using the questionnaire (Section B). The questions were adapted from the Morisky Green Levine Adherence Scale (MGLS) which consists of four composite items with yes/no response options; each ‘yes’ answer was scored ‘1’ and ‘no’ answer was scored ‘0’, giving a score range of 0 to 4. A three level of adherence was suggested based on this score: high (score 0), moderate (score 1–2), and low (score 3–4) (Morisky, Green & Levine, 1986). The Indonesian version of MGLS has been used in a previous study in which acceptable internal consistency was reported (Cronbach’s alpha = 0.64) (Widiyastuti, Setiadi & Wibowo, 2017).

*Lifestyle modifications*. Self-reported information about lifestyle modifications (*i.e.*, salt, diet, physical activity, smoking status and alcohol consumption) were collected using a questionnaire (Section C). This section of the questionnaire was drafted based on previous literature (Warren-Findlow & Seymour, 2011; Akbarpour et al., 2018; Kimani et al., 2019); which was customised for an Indonesian setting. Content validity was checked with an expert panel (*i.e.*, four academic pharmacists in the area of clinical pharmacy and public health) to ensure clarity and the appropriateness with the objectives. This section of the questionnaire was pre-tested on patients with hypertension (*n* = 20) to ensure all questions were understandable.

• *Salt intake behaviour*. Dietary salt intake was assessed with two items: (1) “In the past 7 days, how often did you eat salty foods (such as salted fish, processed meat, instant noodles or salty snack)?”; and (2) “In the past 7 days, how often did you use condiments/seasonings (such as sweet soy sauce, monosodium glutamate, premix seasoning, chili sauce or shrimp/fish paste) in your food?”. The main focus of the questions were to establish general behaviours regarding frequency: never or rarely/occasionally/regularly. While it is difficult to precisely measure salt intake, the questions were developed to explore behaviours that may lead to high salt intake. Based on the household expenditure surveys from Indonesia, the major sources of salt intake are the use of condiments (particularly sweet soy sauce) in addition to sodium obtained from traditional salted products and modern processed foods (Statistics Indonesia, 2013; Statistics Indonesia, 2011; Andarwulan et al., 2011).
• *Physical activity*. Physical activity was assessed by 1 item. “In the past 7 days, how many times did you do physical activity (including what you do around the house or as part of your work)?” A table was provided to list on what day, type of activity on each day, and the duration of each activity. Respondents who reported ≥30 min of moderate-intensity physical activity on 5–7 times in a week was considered ‘adequate’; while less than the requirement was categorised as ‘inadequate’ (Indonesian Society of Hypertension, 2019). The types of activities considered as moderate-intensity physical activity were based on the 2011 Compendium of Physical Activities (Ainsworth et al., 2011).
• *Smoking*. Smoking status was assessed with 1 item, “In the past 7 days, how many times did you smoke a cigarette, even just one puff?” Respondents who reported 0 were considered a ‘non-smoker.’ All others were categorised as a ‘smoker’.
• *Alcohol*. Alcohol intake was assessed with 1 item, “In the past 7 days, how many times did you drink alcohol” Participants who reported 0 were considered as ‘no’ alcohol consumption. All others had ‘yes’ alcohol consumption.
• *Body-weight control*. Body-weight control was determined using Body Mass Index (BMI)—calculated based on weight in kilogram divided by the squared height in metres (kg/m^2^). Patients’ weight and height were measured on a standard beam balance scale with an attached ruler during their visits at the time of the data collection (as recorded in patient records). Based on the guidelines for Asian adults, BMI was classified into four categories: underweight (BMI <18.5), normal weight (18.5 ≤ BMI <23.0), overweight (23.0 ≤ BMI <27.5), and obese (BMI ≥ 27.5) (World Health Organization, 2004).

*BP measurement*. Respondents with a systolic blood pressure of ≤130 mmHg (patients with chronic kidney disease or >65 years: target to 139 mmHg) and a diastolic pressure of ≤79 mmHg diastolic were considered controlled (adapted from InaSH) (Indonesian Society of Hypertension, 2018). Blood pressure was measured using a digital sphygmomanometer during a patient visit at the time of the data collection (as recorded in patient records).

All patients with hypertension visiting the five PHCs were interviewed and examined for the inclusion criteria; in each setting, patients were recruited over a two-week period, during the PHC opening hours. The overall recruitment was conducted between May to October 2019. The data collection was performed after written informed consent was obtained from those willing to participate after a detailed explanation about the nature of the study was provided. Patients were interviewed using the questionnaire by one of five data collectors (*i.e.*, fourth-year pharmacy students). In addition, each of the patient’s record and prescription were reviewed to complete the checklist. A prior briefing session and simulation were performed with the data collectors to ensure that they could collect the data without errors.

### Data analyses

Data were arranged and checked for completeness, coded and entered into Microsoft Excel^®^ and double-checked with the primary sources for accuracy. Descriptive and inferential statistics were generated for each setting as well as in total. Specifically, descriptive data were summarised as frequencies and percentages for categorical data, and mean ± SD for continuous data. Univariate analysis with chi-square was used to test any association with individual adherence factors (*i.e.*, medication adherence and lifestyle modifications—salty food intake, discretionary salt use, physical activity, smoking, alcohol consumption, and body-weight control) with BP control. To explore independent associations of adherence profiles with BP control, a binary logistic regression, that controlled for patient characteristics [*i.e.*, age (≤60; >60 years), gender (female; male), education (none/primary; secondary/university level), occupation (housewife; working—private or public employee/employer; not working/retired), income (<1; ≥1 million IDR), duration of hypertension (≤5 years; >5 years), and presence of comorbidities (yes—DM and/or vascular disease; no)], was performed. Types of antihypertensive therapy were not included because almost all patients received monotherapy (97.5%). All factors were entered into the model and then removed sequentially (stepwise) until those remaining had *p*-values <0.05. Odds ratio (OR) together with 95% confidence interval (CI) more than 1 and *p*-values <0.05 indicated a statistically significant association. IBM SPSS Statistics version 20.0 (IBM Corp, Armonk, NY, USA) was used to assist with the data analysis. 

## Results

Of the 457 patients with hypertension approached in five PHCs in Surabaya, 276 patients consented: PHC A (*n* = 50/91), PHC B (*n* = 65/116), PHC C (*n* = 47/61), PHC D (*n* = 60/88), PHC E (*n* = 54/101), giving response rates of 53–77% and overall 60.4%. The main reasons for refusing participation were lack of time or having other prior appointments.

### Respondent characteristics

The sociodemographic characteristics (*i.e.*, age, gender, level of education, occupation and income) of respondents in each setting and in total are presented in Table 1. The mean age of respondents across the PHCs was more than 55 years, and a majority was female (69.2%) with limited educational attainment (lower secondary or less: 67.4%). About half were housewives, with an income of less than 1 million IDR per month (approximately USD $71). Of those who were employed, most worked in the private sector.

**Table 1:** The characteristics of patients with hypertension in the five PHCs in Surabaya.

**Characteristics**	**PHC A (50)** ***n* (%)**	**PHC B (65)** ***n* (%)**	**PHC C (47)** ***n* (%)**	**PHC D (60)** ***n* (%)**	**PHC E (54)** ***n* (%)**	**Total (276)** **N (%)**
*Age* (mean ± SD, in years)	57.3 ± 10.5	58.8 ± 9.9	60.4 ± 12.4	57.9 ± 11.0	62.9 ± 9.5	59.4 ± 10.7
*Gender*						
Female	34 (68.0)	42 (64.6)	29 (61.7)	48 (80.0)	38 (70.4)	191 (69.2)
Male	16 (32.0)	23 (35.4)	18 (38.3)	12 (20.0)	16 (29.6)	85 (30.8)
*Education*						
None	1 (2.0)	10 (15.4)	4 (8.5)	2 (3.3)	10 (18.5)	27 (9.8)
Primary level	16 (32.0)	35 (53.8)	3 (6.4)	30 (50.0)	9 (16.7)	93 (33.7)
Lower secondary level	9 (18.0)	7 (10.8)	21 (44.7)	14 (23.3)	15 (27.8)	66 (23.9)
Upper secondary level	17 (34.0)	11 (16.9)	5 (10.6)	12 (20.0)	17 (31.5)	62 (22.5)
University level	7 (14.0)	2 (3.1)	14 (29.8)	2 (3.3)	3 (5.6)	28 (10.1)
*Occupation* *						
Not working	2 (4.0)	14 (21.5)	6 (12.8)	1 (1.7)	0 (0.0)	23 (8.3)
Housewife	25 (50.0)	34 (52.3)	25 (53.2)	40 (66.7)	34 (63.0)	158 (57.2)
Civil servant	0 (0.0)	0 (0.0)	2 (4.3)	0 (0.0)	0 (0.0)	2 (0.7)
Private sector employee	12 (24.0)	5 (7.7)	10 (21.3)	8 (13.3)	3 (0.0)	38 (12.8)
Private employer	11 (22.0)	12 (18.5)	1 (2.1)	1 (1.7)	3 (5.6)	28 (10.1)
Others (i.e., retired)	0 (0.0)	0 (0.0)	3 (6.4)	10 (16.7)	14 (25.9)	27 (9.8)
*Personal income (in IDR) (per month)*						
<1 million	5 (10.0)	25 (38.5)	23 (48.9)	39 (65.0)	35 (64.8)	127 (46.0)
1–2 million	17 (34.0)	16 (24.6)	9 (19.1)	13 (21.7)	13 (24.1)	68 (24.6)
2–3 million	14 (28.0)	12 (18.5)	7 (14.9)	4 (6.7)	2 (3.7)	39 (14.1)
>3 million	14 (28.0)	12 (18.5)	8 (17.0)	4 (6.7)	4 (7.4)	42 (15.2)
*Duration of hypertension*						
<1 year	2 (4.0)	4 (6.2)	4 (8.5)	26 (43.3)	12 (22.2)	48 (17.4)
1–5 year	26 (52.0)	34 (52.3)	25 (53.2)	7 (11.7)	0 (0.0)	92 (33.3)
>5 year	22 (44.0)	27 (41.5)	18 (38.3)	27 (45.0)	42 (77.8)	136 (49.3)
*Comorbidities (‘yes’)* ^a^						
Diabetes Mellitus	15 (30.0)	13 (20.0)	1 (2.1)	10 (16.7)	8 (14.8)	47 (17.0)
Vascular disease (CAD, stroke, PVD)	7 (14.0)	3 (4.6)	6 (12.8)	10 (16.7)	12 (22.2)	18 (6.5)
*Family history of hypertension*						
Yes	2 (4.0)	6 (9.2)	0 (0.0)	23 (38.3)	7 (13.0)	38 (13.8)
Don’t know	14 (28.0)	34 (52.3)	29 (61.7)	12 (20.0)	23 (42.6)	112 (40.6)
No	34 (68.0)	25 (38.5)	18 (38.3)	25 (41.7)	24 (44.4)	126 (45.7)
*Antihypertensive drug use*						
*Monotherapy*						
Amlodipine	48 (96.0)	56 (86.2)	47 (100.0)	54 (90.0)	41 (75.9)	246 (89.1)
Nifedipine	0 (0.0)	1 (1.5)	0 (0.0)	3 (5.0)	0 (0.0)	4 (1.4)
Hydrochlorothiazide	0 (0.0)	1 (1.5)	0 (0.0)	0 (0.0)	0 (0.0)	1 (0.4)
Captopril	0 (0.0)	1 (1.5)	0 (0.0)	3 (5.0)	13 (24.1)	17 (6.2)
Bisoprolol	0 (0.0)	1 (1.5)	0 (0.0)	0 (0.0)	0 (0.0)	1 (0.4)
*Combination therapy*						
Amlodipine + Hydrochlorotiazide	2 (4.0)	5 (7.7)	0 (0.0)	0 (0.0)	0 (0.0)	7 (2.5)

**Notes.**

Abbreviations PHC Primary Health Centre CAD coronary artery disease PVD peripheral vascular disease IDR Indonesian Rupiah SD standard deviation

a Frequencies and percentages of patients who had the comorbidity (‘yes’) as recorded in the medical record.

With regards to clinical factors, approximately 50% of the respondents were diagnosed with hypertension more than 5 years ago, and had reported no family history of hypertension. Approximately 20% of respondents were recorded in the patient medical record with related vascular disease or diabetes.

### Antihypertensive drug use

The profile of medication use for hypertension is summarised in Table 1. Across the five PHCs 92.3% to 100.0% were prescribed a single drug, with amlodipine most commonly prescribed, with a dose of 5 to 10 mg once daily.

### Adherence to medication and lifestyle modification

Adherence to antihypertensive medications and healthy lifestyle practices across the five PHCs and overall are presented in Table 2. More than half of the respondents did not take medication as prescribed (low to intermediate adherence 65.2%). A majority of respondents reported to rarely eat salty food (56.2%), had never smoked (76.8%) or consumed alcohol (99.6%), and had normal body mass index (57.2%). However, most of the respondents reported inadequate physical activity (87.7%) and about half reported regular addition of salt to food (50.4%).

**Table 2:** Adherence to medication and healthy behaviours among hypertensive patients in the five PHCs.

	**PHC A (50)** ***n* (%)**	**PHC B (65)** ***n* (%)**	**PHC C (47)** ***n* (%)**	**PHC D (60)** ***n* (%)**	**PHC E (54)** ***n* (%)**	**Total (276)** **N (%)**
**Medication adherence**						
High	5 (10.0)	28 (43.1)	13 (27.7)	28 (46.7)	22 (40.7)	96 (34.8)
Intermediate	36 (72.0)	24 (36.9)	21 (44.7)	22 (36.7)	21 (38.9)	124 (44.9)
Low	9 (18.0)	13 (20.0)	13 (27.7)	10 (18.7)	11 (20.4)	56 (20.3)
**Lifestyle** **modification** ^a^						
*Salt intake behaviour*						
-Salty food intake						
Never or rarely	31 (62.0)	42 (64.6)	28 (59.6)	30 (50.0)	24 (44.4)	155 (56.2)
Occasionally	11 (22.0)	10 (15.4)	16 (34.0)	16 (26.7)	25 (46.3)	78 (28.3)
Regularly	8 (16.0)	13 (20.0)	3 (6.4)	14 (23.3)	5 (9.3)	43 (15.6)
-Discretionary salt use						
Never or rarely	17 (34.0)	3 (4.6)	23 (48.9)	9 (15.0)	37 (68.5)	89 (32.2)
Occasionally	16 (32.0)	4 (6.2)	10 (21.3)	4 (25.9)	14 (29.2)	48 (17.4)
Regularly	17 (34.0)	58 (89.2)	14 (29.8)	47 (78.3)	3 (5.6)	139 (50.4)
*Physical activity*						
Adequate	3 (6.0)	5 (7.7)	17 (36.2)	2 (3.3)	7 (13.0)	34 (12.3)
Inadequate	47 (94.0)	60 (92.3)	30 (63.8)	58 (96.7)	47 (87.0)	242 (87.7)
*Smoking*						
Non-smoker	34 (68.0)	50 (76.9)	31 (66.0)	58 (96.7)	39 (72.2)	212 (76.8)
Smoker	16 (32.0)	15 (23.1)	16 (34.0)	2 (3.4)	15 (27.8)	64 (23.2)
*Alcohol consumption*						
No	49 (98.0)	65 (100.0)	47 (100.0)	60 (100.0)	54 (100.0)	275 (99.6)
Yes	1 (2.0)	0 (0.0)	0 (0.0)	0 (0.0)	0 (0.0)	1 (0.4)
*Body-weight control*						
Underweight	1 (2.0)	2 (3.1)	2 (4.3)	3 (5.0)	3 (5.6)	11 (4.0)
Normal weight	24 (48.0)	32 (49.2)	26 (55.3)	33 (55.0)	43 (79.6)	158 (57.2)
Overweight	7 (14.0)	11 (16.9)	6 (12.8)	9 (15.0)	8 (14.8)	41 (14.9)
Obese	18 (36.0)	20 (30.8)	13 (27.7)	15 (25.0)	0 (0.0)	66 (23.9)

**Notes.**

Abbreviations PHC Primary Health Centre CI confidence interval

a Salt intake behaviour (frequency of salty food consumption: never/rarely, occasionally, regularly; frequency of discretionary salt use: never/rarely, occasionally, regularly); physical activity (inadequate: <30 minutes of moderate intensity activity 5 times per week, adequate: ≥30 min of moderate intensity activity 5–7 times per week); smoking (non-smoker: 0 puff, smoker: any others); alcohol consumption (no: 0 consumption; yes: any others); body-weight control [assessed using Body Mass Index (BMI): underweight (BMI <18.5), normal weight (18.5 ≤BMI <23.0), overweight (23.0 ≤ BMI <27.5), and obese (BMI ≥ 27.5)].

### Blood pressure measurements in respondents

Among the respondent’s blood pressure measurements, only 31.9% had their blood pressure controlled. There were wide disparities in BP control across PHCs, with the highest level occurring in PHC A (62.0%) and the lowest in PHC D (15.0%) (Table 3).

**Table 3:** Blood pressure control among patients with hypertension in the five PHCs.

**Blood pressure** ^a^	**PHC A (50)** ***n* (%)**	**PHC B (65)** ***n* (%)**	**PHC C (47)** ***n* (%)**	**PHC D (60)** ***n* (%)**	**PHC E (54)** ***n* (%)**	**Total (276)** **N (%)**	Mean (95% CI) Systolic blood pressure (mmHg)	Mean (95% CI) Diastolic blood pressure (mmHg)
Controlled	31 (62.0)	11 (16.9)	8 (17.0)	9 (15.0)	29 (53.7)	88 (31.9)	122.5 (120.8–123.9)	78.2 (77.2–79.0)
Not controlled	19 (38.0)	54 (83.1)	39 (83.0)	51 (85.0)	25 (46.3)	188 (68.1)	145.5 (143.5–147.4)	90.0 (88.9–91.1)

**Notes.**

Abbreviations PHC Primary Health Centre

a Successful blood pressure control was defined as ≤130 mm Hg systolic and ≤79 mm Hg diastolic (unless for patients with chronic kidney disease or >65 years,the systolic target could increase to 139 mm Hg).

Associations between adherence profiles and BP control across the five PHCs are presented in Table 4. Univariate analysis of individual adherence factors indicated that medication adherence, discretionary salt use and smoking status had significant associations with BP control (all *p*-values ≤0.05). A binary logistic regression—after controlling for patient characteristics (*i.e.*, age, gender, education, occupation, income, duration of treatment, duration of diabetes and presence of comorbidities)—confirmed these findings. Patients with high/intermediate medication adherence were 2.13 times more likely to have controlled BP compared to those with low adherence (95% CI [1.02–4.46], Wald *χ*2(1) = 4.02, *p* < 0.045); while the odds of non-smokers and less discretionary salt use having controlled BP were 2.99 (95% CI 1.45 to 6.21, Wald *χ*2(1) = 8.76, *p* < 0.003) and 2.51 (95% CI 1.44 to 4.39, Wald *χ*2(1) = 10.40, *p* < 0.001) times compared to non-smokers and those with regular salt use, respectively. Detailed results of the univariate analysis and logistic regression are reported in Table 4. The correlation between blood pressure control and alcohol consumption was not tested as most patients did not drink alcohol.

**Table 4:** Associations between adherence and blood pressure control.

	**PHC A (50)** **Blood Pressure**		**PHC B (65)** **Blood Pressure**		**PHC C (47)** **Blood Pressure**		**PHC D (60)** **Blood Pressure**		**PHC E (54)** **Blood Pressure**		**Total (276)Blood Pressure** (univariate analysis)			**Total (276)Blood Pressure** (multivariate analysis)	
	Controlled *n* (%)	Not controlled *n* (%)	Controlled *n* (%)	Not controlled *n* (%)	Controlled (%)	Not controlled *n* (%)	Controlled *n* (%)	Not controlled *n* (%)	Controlled *n* (%)	Not controlled *n* (%)	Controlled *n* (%)	Not controlled *n* (%)	*p*-value	Controlled	*p*-value
**Medication adherence**															
High/intermediate	28 (90.3)	13 (68.4)	9 (81.8)	43 (79.6)	6 (75.0)	28 (71.8)	8 (88.9)	42 (82.4)	27 (93.1)	22 (88.0)	77 (87.5)	143 (76.1)	0.028^a^	2.13 (1.02–4.64)	0.045^b^
Low	3 (9.7)	6 (31.6)	2 (18.2)	11 (20.4)	2 (25.0)	11 (28.2)	1 (11.1)	9 (17.6)	2 (6.9)	3 (12.0)	11 (12.5)	45 (23.9)		reference	
**Lifestyle modification**															
Salt intake behaviour															
-Salty food intake															
Never or rarely/occasionally	25 (80.6)	17 (89.5)	8 (72.7)	44 (81.5)	8 (100.0)	36 (92.3)	8 (88.9)	38 (82.6)	27 (93.1)	22 (88.0)	76 (86.4)	157 (83.5)	0.597	–	
Regularly	6 (19.4)	2 (10.5)	3 (23.1)	10 (18.5)	0 (0.0)	3 (7.7)	1 (11.1)	13 (25.5)	2 (6.9)	3 (12.0)	12 (13.6)	31 (16.5)			
-Discretionary salt use															
Never or rarely/occasionally	20 (64.5)	13 (68.4)	2 (18.2)	5 (9.3)	3 (37.5)	30 (76.9)	4 (44.4)	9 (17.6)	26 (89.7)	25 (100.0)	55 (62.5)	82 (43.6)	0.004^a^	2.51 (1.44–4.39)	0.001^b^
Regularly	11 (35.5)	6 (31.6)	9 (81.8)	49 (90.7)	5 (62.5)	9 (23.1)	5 (55.6)	42 (82.4)	3 (10.3)	0 (0.0)	33 (37.5)	106 (56.4)		reference	
Physical activity															
Adequate	3 (9.7)	0 (0.0)	2 (18.2)	3 (5.6)	4 (50.0)	13 (33.3)	0 (0.0)	2 (3.9)	2 (6.9)	5 (20.0)	11 (12.5)	23 (12.2)	0.950	–	
Inadequate	28 (90.3)	19 (100.0)	9 (81.8)	51 (94.4)	4 (50.0)	26 (66.7)	9 (100.0)	49 (96.1)	27 (93.1)	20 (80.0)	77 (87.5)	165 (87.8)			
Smoking															
Non-smoker	26 (83.9)	8 (42.1)	9 (81.8)	41 (75.9)	7 (87.5)	24 (61.5)	8 (88.9)	50 (98.0)	24 (82.8)	15 (60.0)	74 (84.1)	138 (73.4)	0.050^a^	2.99 (1.45–6.21)	0.003^b^
Smoker	5 (16.1)	11 (57.9)	2 (18.2)	13 (24.1)	5 (12.5)	15 (38.5)	1 (11.1)	1 (2.0)	5 (17.2)	10 (40.0)	14 (15.9)	50 (26.6)		reference	
Alcohol consumption															
No	31 (100.0)	18 (94.7)	11 (100.0)	54 (100.0)	8 (100.0)	39 (100.0)	9 (100.0)	51 (100.0)	29 (100.0)	25 (100.0)	0 (0.0)	1 (0.5)	N/A	–	
Yes	0 (0.0)	1 (5.3)	0 (0.0)	0 (0.0)	0 (0.0)	0 (0.0)	0 (0.0)	0 (0.0)	0 (0.0)	0 (0.0)	88 (100.0)	187 (99.5)			
Body-weight control															
BMI < 23	16 (51.6)	9 (47.4)	8 (72.7)	26 (48.1)	4 (50.0)	24 (61.5)	7 (77.8)	29 (56.9)	26 (89.7)	20 (80.0)	61 (69.3)	108 (57.4)	0.059	–	
BMI ≥ 23	15 (48.4)	10 (52.6)	3 (27.3)	28 (51.9)	4 (50.0)	15 (38.5)	2 (22.2)	22 (43.1)	3 (10.3)	5 (20.0)	27 (30.7)	80 (42.6)			

**Notes.**

Abbreviations PHC Primary Health Centre BMI Body Mass Index N/A not available OR odds ratio CI confidence interval

a Significant associations from univariate analysis (chi-square tests).

b Significant associations from multivariate analysis (binary logistic regression—stepwise method) after adjusted for patient characteristics (i.e., age, gender, education, occupation, income, duration of hypertension, and presence of comorbidities).

## Discussion

This study has provided current evidence related to the management of patients with hypertension across primary care clinics in Surabaya, Indonesia. Most patients were treated with a single antihypertensive medication, but importantly had not achieved blood pressure targets based on the recent 2019 InaSH consensus statements. There were wide disparities in BP control as well as adherence profiles among hypertensive patients across PHCs. The regression analysis has identified low medication adherence, discretionary salt use and smoking as three factors independently associated with uncontrolled blood pressure. This finding importantly identifies medication adherence as an important factor, together with regular addition of salt and smoking as factors that should be included in planned interventions to improve patient outcomes.

It should be noted that this study was conducted during the period when the 2013 National Formulary was current and has been analysed accordingly. From April 2020, lisinopril was added in ACEI class (Ministry of Health Republic of Indonesia, 2019), thus increasing prescribing options.

A majority of patients was female (69.2%) and many were housewives (57.2%). Less than one-third were working reflecting a mean age above 55 years. This is as expected, as high blood pressure usually occurs from 55 years in women and 45 years among men (Pearson et al., 1997). However, the Indonesia Family Life Survey (IFLS) in 2016 reported that hypertension prevalence was only slightly higher in women than men (52.3% versus 43.1%) (Hussain et al., 2016), which raises concerns that females might access PHCs to a greater extent than males. Older males were reportedly more physically active than females (Arifin, Braun & Hogervorst, 2021); possibly conflicting with the opening hours of PHCs’ outpatient services from 07.30 am to 14.00 pm Monday to Saturday. Additionally, the short period of medication provision (usually two weeks to one month) requires patients to attend the PHCs very frequently for repeat medications.

Further, the overall low educational background of the patients may indicate a lack of understanding of risk factors, for the prevention and management of hypertension (Kimani et al., 2019). False impressions, such as ‘taking antihypertensive medications should be discontinued once blood pressure is controlled,’ and being afraid of side effects, are reported to cause drug discontinuation (Kimani et al., 2019; Tan et al., 2017). A higher level of education was associated with higher socioeconomic status, which is often linked to disease awareness and health-seeking behaviours (Mills et al., 2016). Hence, the low educational background among patients with hypertension in these Indonesian PHCs warrants the development of appropriate educational strategies whilst considering the already high workload among health professionals in PHCs. The high workload is partially created by frequent visits of patients many of whom are stabilised to renew their prescription. Optimising the use of community pharmacies for medication refills could be an option. This also improves accessibility as most community pharmacies have longer opening hours, and some even provide a 24 hour-service. In addition to medication supply, as the first-point of contact, community pharmacists are in an ideal position to provide education to ensure quality use of medicines (Rahmawati & Bajorek, 2018; Wulandari et al., 2021; Setiadi et al., 2020).

The poor levels of medication adherence among patients with hypertension in this study warrants further attention (low/intermediate level 65.2%). The logistic regression indicated medication adherence as one of the significant factors contributing to poor BP control. The poor adherence found in this study is comparable to findings from a wide range of countries reporting medication non-adherence in controlled hypertensive patients of 59.7% and in uncontrolled 83.7%, although there were wide confidence intervals involved in these reports (Abegaz et al., 2017). A large European study reported that adherence to hypertension treatment decreased over time; which was confirmed in a follow-up 10-year study where more patients discontinued taking their antihypertensive medication every year of the study (Mazzaglia et al., 2005; Van Wijk et al., 2005). A Kenyan study has reported that many people were more familiar with the treatment of communicable diseases where once the problem was controlled the medication could be stopped (Kimani et al., 2019). While approximately 50% of patients in this study had hypertension diagnosed for more than five years, the healthcare system should ensure patient awareness of the chronic nature of hypertension and improve efforts in understanding that adherence to hypertension treatment helps achieving optimal health outcomes. It should however be noted that these patients were attending the PHCs and there would be other groups that had either ceased attending or never attended.

There are wide disparities in BP control across PHCs that might be related to varied lifestyle behaviours among those attending PHCs. In general, lifestyle modification profiles were better in the BP controlled group than in the uncontrolled group across the five PHCs (Table 4). The logistic regression further indicated that discretionary salt use and smoking status were significant factors that contributed to poor BP control. The relationship between hypertension and dietary sodium intake is widely recognised (Grillo et al., 2019). Encouragingly, smoking reported in this study was low and almost no alcohol was consumed. This might be due to the fact that the majority of Indonesians are muslims and share Islamic dietary laws that prohibits alcohol intake (Kimani et al., 2019). Additionally, the majority of participants were women who in Indonesian culture are distanced from smoking and consuming alcohol (Kimani et al., 2019). However, most men in this study were smokers which warrants further consideration for effective smoking cessation programs, or even prevention programs as part of national health campaigns. The low physical activity reported across the PHCs also requires appropriate interventions.

There were several strengths and limitations to this study. It used a census sample in the five selected PHCs, which were located in different socioeconomic areas across Surabaya, achieving an overall response rate of 60.4%. Although a sound response rate was achieved, there is a possibility that non-responders did not share the same profile, thus some caution should be exercised in generalising the findings. Additionally, the data might suffer from some recall bias as patients were asked to remember health behaviour actions. However, the questions were designed for patients to recall their behaviours within a short period of time (the last 7 days), thus recall bias was expected to be reduced. Medication adherence was self-reported using a validated questionnaire, but not verified by biological markers. Moreover, this study did not assess some important determinants of high blood pressure, such as food intake or stress level. However, this study provides important patient data to guide the development of patient- and population-based strategies for better hypertension management in Indonesian primary care settings.

## Conclusions

Additional strategies to improve blood pressure control among patients with chronic hypertension in these Indonesian primary care settings are essential. In addition to quality use of medicines, healthy lifestyle practices should be emphasised as part of the treatment plan; salt reduction behaviour and quitting smoking have tended to have positive impacts on blood pressure control. Community pharmacists could contribute by dispensing medications at monthly intervals for patients whose hypertension is controlled and who may only require three to six monthly medical consultations.

## Supplemental Information

10.7717/peerj.13171/supp-1 Supplemental Information 1 Data collection tool (questionnaire and checklist)

10.7717/peerj.13171/supp-2 Supplemental Information 2 Raw Data Hypertension in Indonesia primary care settings and codebook

## Additional Information and Declarations

### Data Availability

The following information was supplied regarding data availability: The raw data measurements are available in the Supplementary File.
