# Supplementary material for: Knowing the gap: medication use, adherence and blood pressure control among patients with hypertension in Indonesian primary care settings
Source: PeerJ. 2022 Mar 25;10:e13171. doi: 10.7717/peerj.13171 (PMC8958961; doi:10.7717/peerj.13171)
Supplement: Supplemental Information 1 [file peerj-10-13171-s001.pdf]

DATA COLLECTION TOOL

**SECTION A: Patient characteristics**			
**Sociodemographic**<br>**(Note: data collected from patient interview)**			
Year of birth:		…………………	
Gender:		☐ Male<br>☐ Female	
Level of education:		☐ No<br>☐ Primary level<br>☐ Lower secondary level<br>☐ Upper secondary<br>☐ University level	
Occupation:		☐ Housewife<br>☐ Civil servant<br>☐ Private sector employee<br>☐ Private employer<br>☐ Not working	
Income:		☐ <1 million IDR<br>☐ 1-2 million IDR<br>☐ 2-3 million IDR<br>☐ >3 million IDR	
**Antihypertensive drug use**<br>**(Note: data collected from patient prescription)**			
**Name of antihypertensive drug**	**Dose**	**Frequency**	
1……………………<br>2…………………..<br><br>*(add more rows when needed)*	…………………..<br>…………………..	…………………..<br>…………………..	
**SECTION B: Adherence**<br>**(Note: data collected from patient interview)**			
Q1: Do you ever forget to take your hypertensive medication?		☐ Yes	☐ No
Q2: Do you ever have problems remembering to take your hypertensive medication?		☐ Yes	☐ No
Q3: When you feel better, do you sometimes stop taking your hypertensive medication?		☐ Yes	☐ No
Q4: Sometimes if you feel worse when you take your hypertensive medication, do you stop taking it?		☐ Yes	☐ No
**SECTION C: Lifestyle modifications**			
**Salt intake behaviour**<br>**(Note: data collected from patient interview)**			
Q1: "In the past 7 days, how often did you eat salty foods (such as salted fish, processed meat, instant noodles or salty snack)?"		☐ Never/rarely<br>☐ Occasionally	

	☐ Regularly
Q2: "In the past 7 days, how often did you use condiments/seasonings (such as sweet soy sauce, monosodium glutamate, premix seasoning, chili sauce or shrimp/fish paste) in your food?"	☐ Never/rarely<br>☐ Occasionally<br>☐ Regularly
**Physical activity**<br>**(Note: data collected from patient interview)**	
Q1: In the past 7 days, how many times did you do physical activity (including what you do around the house or as part of your work)?	

**Day**	**Type of Activity**	**Duration**
1	1……………………………..<br>2 …………………………….<br>……………………………….<br>*(add more rows when needed)*	…………. min<br>…………. min<br>…………. min
2		
3		
4		
5		
6		
7		

**Smoking**<br>**(Note: data collected from patient interview)**	
Q1: In the past 7 days, how many times did you smoke a cigarette, even just one puff?	……………………… times
**Alcohol**<br>**(Note: data collected from patient interview)**	
Q1: In the past 7 days, how many times did you drink alcohol?	……………………… times
**Body Mass Index (BMI)**<br>**(Note: data collected from patient record)**□	
Height: ………………… metres<br>Weight: …………………. kilograms<br>BMI = weight (kg)/ height (m)^2	……………………… kg/m^2
**BP measurement**	
Diastole:<br>Systole :<br>**(Note: data collected from patient record)**	……………………. mmHg<br>……………………. mmHg
